# Rare genetic variants provide a mechanistic basis for immune imbalance in IgG4-related disease

**DOI:** 10.1172/JCI183396

**Published:** 2024-08-15

**Authors:** Dominic J. Ciavatta

**Affiliations:** Department of Genetics, UNC Kidney Center, Division of Nephrology and Hypertension, Department of Medicine, Chapel Hill, North Carolina, USA.

## Abstract

Autoimmune diseases are commonly associated with a polygenic inheritance pattern. In rare instances, causal monogenic variants have been identified. The study by Liu et al. in this issue of the *JCI* provides an example of monogenic variants occurring in patients with IgG4-related disease (IgG4-RD). The authors investigated a familial cluster of IgG4-RD that consisted of an affected father and two daughters; the mother was unaffected. Genome sequencing of this quad identified a variant in *IKZF1* (encoding IKAROS) and another variant in *UBR4* (encoding E3 ubiquitin ligase). Both variants were present in the father and both daughters but absent in the unaffected mother. Using multidimensional profiling of immune cells and functional experiments in primary cells, the authors determined a molecular pathway contributing to T cell activation in IgG4-RD. Importantly, the characterization of these variants provides insights into pathogenic mechanisms in IgG4-RD and, potentially, other autoimmune diseases.

## IgG4-RD and potential mechanism of pathogenesis

IgG4-related disease (IgG4-RD) is a rare immune-mediated fibroinflammatory condition associated with high serum IgG4 levels and infiltration of IgG4^+^ plasma cells in affected organs (1). Common organs affected include the pancreas, the lacrimal and salivary glands, the retroperitoneum, and the lymph nodes. A correct diagnosis to distinguish IgG4-RD from malignancy requires results from clinical, radiological, and histological tests. Diagnostic histological characteristics of lesions include storiform (swirling) fibrosis and lymphoplasmacytic infiltration causing vascular inflammation with obstructive clotting (2). These destructive lesions can lead to organ failure if left untreated. Initial treatment to induce remission typically involves glucocorticoids, followed by steroid-sparing immunosuppressive drugs such as azathioprine or cyclophosphamide. Biologics, such as rituximab, have been used to maintain remission (3). Although these therapeutic strategies are successful for treating IgG4-RD, the fact that these therapies also benefit other inflammatory and autoinflammatory conditions highlight the incomplete understanding of the pathogenic mechanisms specific to IgG4-RD.

Although the underlying pathogenesis in IgG4-RD is unclear, molecular features of the disease support the current Th2-centric hypothesis (4), in which increased IgG4 reflects class switching rather than the production of disease-initiating autoantibodies. This idea suggests that Th2 cytokines such as IL-4 and IL-13 promote B cell production and class switching of IgE and IgG4. Indeed, increased production of these Th2 cytokines, as well as of IL-5 and IL-10, have been reported in IgG4-RD (5). In addition, the common atopic manifestations characteristic of many patients diagnosed with IgG4-RD implicates a Th2 response (6). In this issue of the *JCI*, Liu et al. (7) describe a genetic basis for a proallergic immune imbalance in patients with IgG4-RD.

## Genetic basis for Th2 polarization

Genome sequencing of a family consisting of a father and two daughters diagnosed with IgG4-RD and an unaffected mother identified potential candidate variants (7). The father and both daughters, but not the mother, carried two rare variants not annotated in the gnomAD database. One was a missense variant in *IKZF1*, which encodes the transcription factor IKAROS, at position 548G>A. The resulting amino acid substitution (Arg183His) in the DNA binding domain was predicted to be a gain-of-function (GOF) variant. *IKZF1* variants in the DNA binding domain have been identified previously through exome sequencing of four unrelated families with autoimmune, allergic, and lymphoproliferative conditions (8). The other variant was a nonsense variant in *UBR4*, which encodes an E3 ubiquitin ligase at position 12537T>A and was predicted to result in a truncated protein (Cys4179Ter [UBR4-C4179Ter]) (7).

To assess the affect of these variants on immune cell populations from the three patients and three healthy individuals, PBMCs were immunophenotyped by mass cytometry (CyTOF). While there were some differences in the frequencies of immune cell subsets, the most striking finding was a reproducible increase in the levels of CD45 (a membrane tyrosine phosphatase, encoded by *PTPRC*) on all immune cells, particularly T cells (7). CD45 regulates T cell receptor (TCR) signaling by dephosphorylating intracellular signaling molecules, and increased phosphatase activity from elevated CD45 is predicted to downregulate TCR signaling via reduced phosphorylation of the Src family protein tyrosine kinase LCK (9–11). Although the authors detected reduced LCK phosphorylation in T cells from the patients, phosphorylation of an LCK downstream target, ZAP70, was increased. To test whether another Src family member was responsible for enhanced TCR signaling in the patients, the authors measured FYN, a tyrosine kinase involved in TCR signaling, and detected elevated protein levels in T cells from the patients. FYN expression was also elevated in EBV-transformed B cells from the patients. Results from overexpression of FYN in T cells from healthy individuals and siRNA-mediated knockdown of FYN in T cells from the three patients demonstrated a requirement of FYN for T cell signaling and activation (7). Interestingly, CD45 acts only on FYN’s inhibitory phosphorylation site (Y527) (12), and testing T cells from the patients revealed reduced phosphorylation only at Y527. These results focused attention on FYN as a primary regulator of T cell activation in IgG4-RD.

**Figure 1:**
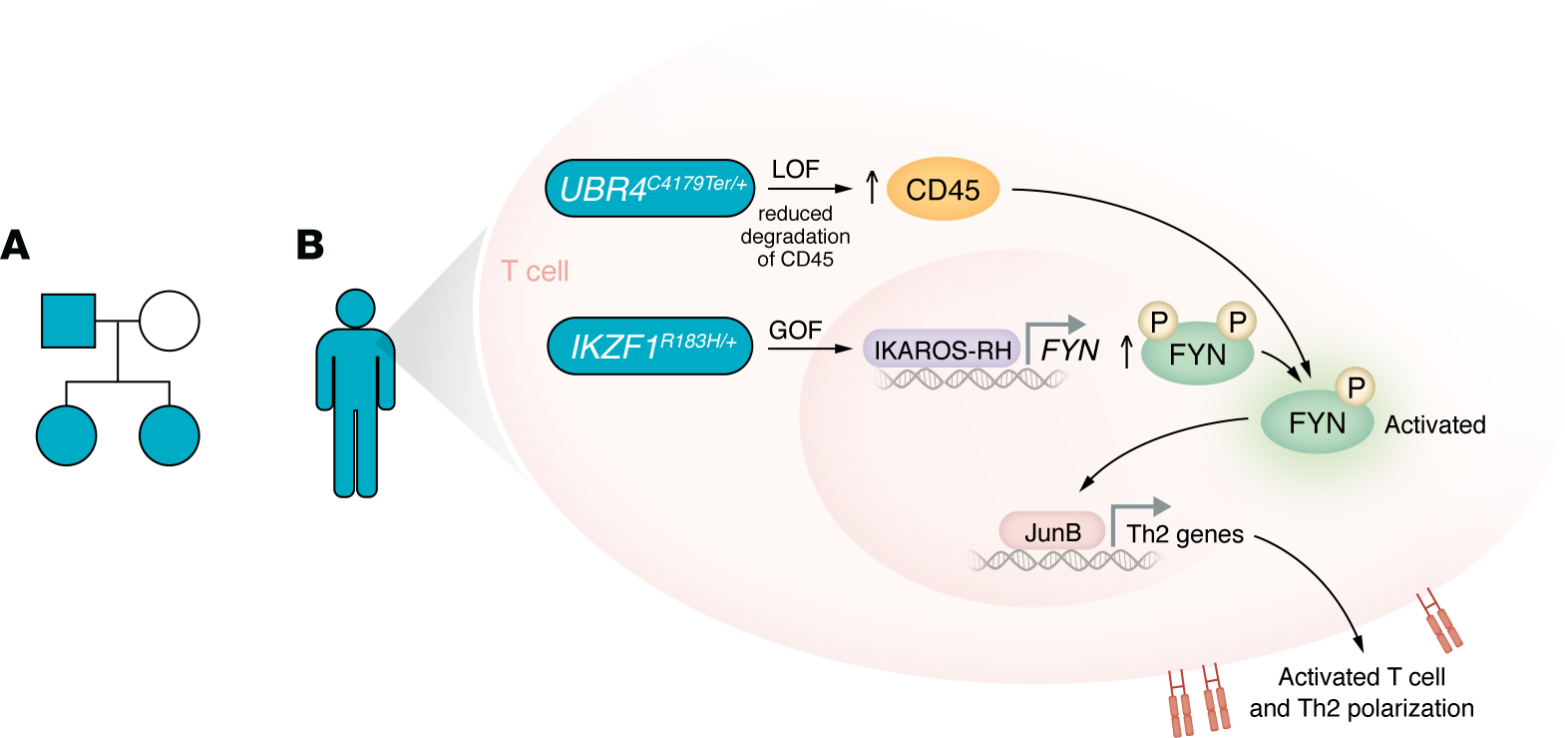
Patients with IgG4-RD possess genetic variants responsible for Th2 polarization and T cell activation. (**A**) Three members of a family were affected by IgG4-RD: the father (teal square) and two daughters (teal circles). The mother was unaffected (white circle). (**B**) Rare variants were detected in the genome sequence of the affected individuals. The GOF IKAROS-R183H variant increases FYN expression, and the LOF UBR4-C4179Ter variant inhibits clearance of the tyrosine phosphatase CD45, which removes inhibitory phosphate, sparing phosphorylation at the activating tyrosine residue and leading to activation of FYN. The consequence of these variants for FYN stabilizes JunB to drive Th2 response genes and T cell activation.

The next question Liu and colleagues addressed explored the cause of dysregulated protein levels: Were FYN and CD45 dysregulated transcriptionally or posttranscriptionally in the patients? By measuring gene expression of *FYN* and *PTPRC* (encoding CD45), the authors discovered an increased *FYN*, but not *PTPRC*, message, indicating transcriptional control of FYN and posttranscriptional control of CD45. The authors tested the association of increased FYN and CD45 with variants in *IKZF1* and *UBR4*. Knockdown of *IZKF1* resulted in reduced FYN with no change in CD45, whereas knockdown of *UBR4* increased CD45 with no change in FYN. A direct mechanism through transcriptional control of FYN expression by the *IKZF1* variant was demonstrated with ChIP experiments and luciferase reporter assays (7). IKAROS-R183H bound the *FYN* promoter in primary T cells and increased luciferase expression driven by the *FYN* promoter, confirming this variant’s GOF activity (8). Investigating a potential role of UBR4 truncation in posttranscriptional regulation of CD45, the authors blocked lysosomal protein degradation, which, similar to *UBR4* knockdown, stabilized CD45. These data support the authors’ conclusion that increased FYN activity results from IKAROS-R183H driving increased expression of *FYN*; and UBR4 truncation stabilizes CD45, which removes phosphorylation at the inhibitory Y527 site of FYN (7) (Figure 1).

The authors investigated the effect of this synergistic effect on FYN activity as it relates to T cell activation. Using transgenic expression of a low-avidity, self-reactive TCR that recognizes an islet-specific autoantigen (glucose-6 phosphate catalytic subunit–related protein), they showed that T cells from the patients with the *IKZF1* GOF and *UBR4* truncation variants had a lower threshold for T cell activation compared with T cells from healthy individuals. These data support the existence of a mechanism for self-reactive T cells to break tolerance, whereby increased FYN activity enhances TCR signaling (7).

A hallmark of IgG4-RD is the differentiation of immune cells into Th2 cells. CyTOF data indicated that the patients had increased Th2 and decreased Th1 cell frequencies. In addition, transcripts for the Th2 cytokines IL-4, IL-5, and IL-13 were upregulated in the three patients, while the Th1 and Th17 cytokines IFN-γ (*INFG*) and IL-17 (*IL17*) were decreased. Overexpression of FYN in T cells from healthy individuals was sufficient to promote IL-4 production — importantly, without affecting *INFG* or *IL17* mRNA. Evidence that FYN was capable of inducing Th2 polarization in vivo came from mouse experiments, in which CD4^+^ T cells were transduced to overexpress FYN and transferred into mice followed by ovalbumin immunization. This immunization induced Th2 differentiation in mice that had overexpression of FYN (7).

After establishing the importance of FYN in skewing toward Th2 differentiation, Liu et al. (7) investigated a mechanism through which FYN may function. The E3 ubiquitin ligase ITCH is regulated by FYN. FYN phosphorylation of ITCH reduces the ubiquitination of JunB by ITCH, thus stabilizing JunB (13). Indeed, JunB was elevated in the patients’ T cells, and overexpression of FYN in T cells from healthy individuals resulted in higher JunB levels. As a final support for their proposed mechanism for a genetic basis of Th2 polarization, the authors showed that T cells expressing the IKAROS-R183H variant compared with WT IKAROS had elevated FYN and JunB in conjunction with increased expression of the Th2 lineage transcription factor *GATA3* (7).

## Future directions

The genetic findings and functional studies of Liu et al. (7) are an important advance in understanding the mechanism for a Th2 bias in patients with IgG4-RD and allergy and/or atopy. The GOF IKAROS-R183H variant increased the expression of FYN, and simultaneously, the loss-of-function (LOF) variant UBR4-C4179Ter disrupted lysosome-mediated degradation of CD45, which activated FYN. Activated FYN stabilized JunB, thereby inducing the expression of Th2 response genes (Figure 1). While previous studies implicated IKAROS deficiency in contributing to autoimmunity (14), the results presented here (7), as well as the identification of GOF IKAROS variants in individuals with inflammatory conditions (8), indicate a more complicated picture of IKAROS function. Profiling the regulatory landscape of IKAROS GOF variants could identify additional pathways contributing to T cell activation and potential biomarkers to monitor the response to therapy. More broadly, these findings open the possibility that IKAROS plays a role in other atopic diseases (7). The discovery of defective protein degradation mediated by the UBR4 variant suggests that regulation of protein turnover also contributes to autoimmunity. As Liu and authors point out, profiling the proteome in patients who are deficient for protein degradation could identify biomarkers and pathways involved in the inflammatory response (7). The findings of Liu et al. suggest that defining the IKAROS GOF genomic-binding landscape or profiling the proteome will uncover synergistic mechanisms that modulate immune function.

## Version 1 08/15/2024

Electronic publication
